# Short Term Endpoints for Cancer Screening Trials: Does Tumor Subtype Matter?

**DOI:** 10.1158/1055-9965.EPI-22-1307

**Published:** 2023-06-01

**Authors:** Lukas Owens, Kemal Caglar Gogebakan, Usha Menon, Roman Gulati, Noel S Weiss, Ruth Etzioni

**Affiliations:** 1 Division of Public Health Sciences, Fred Hutchinson Cancer Center; 2 Center for Early Detection Advanced Research, Knight Cancer Institute; 3 Institute of Clinical Trials and Methodology, University College London; 4 Department of Epidemiology, University of Washington

## Abstract

Multi-cancer early detection tests are precipitating a re-examination of potential short-term endpoints for cancer screening trials. A reduction in advanced stage incidence is a prime candidate, and stage-shift models that substitute early-stage for late-stage survival have been used to predict mortality reduction due to screening. However, standard stage-shift models often ignore prognostic subtypes, effectively implying that cancers detected early also have an associated subtype shift. To illustrate the differences between mortality predictions from stage-shift models that ignore versus preserve prognostic subtype, we use ovarian cancer partitioned by histologic subtype and prostate cancer partitioned by grade. We infer general conditions under which stage-shift models that preserve prognostic subtype are likely to predict mortality reductions that differ from those that ignore subtype and examine the implications for short-term endpoints based on stage in cancer screening trials.

The cancer early detection endeavor is based on the expectation that advancing diagnosis to a point at which a tumor is amenable to curative treatment should lead to an extension of cancer-specific survival. This is frequently formalized via a *stage-shift model* that assumes cases detected and treated at an earlier stage secure a corresponding increase in their disease-specific life expectancy [1, 2, 3].

The stage-shift model implies that a screening test’s impact on disease-specific mortality can be predicted using only information available at the time of diagnosis. This early prediction system is now of great interest in considering the next generation of cancer screening trials. Conventional trials require many years of follow-up to determine how screening impacts the established primary endpoint of cancer-specific mortality. With a pipeline of new cancer screening tests including multi-cancer early detection tests, there is an urgent need for trials that can expeditiously inform about screening benefit. If the stage-shift model holds, such trials could, in principle, be designed to evaluate mortality benefit using an incidence-based endpoint, which could shorten trial duration and reduce the required sample size considerably. In fact, the primary endpoint in an ongoing trial of a multi-cancer early detection test in the United Kingdom is the reduction in the incidence of late-stage diagnoses [4].

Although the stage-shift model is intuitive, it has important caveats. First, a given reduction in late-stage incidence does not generally imply a similar reduction in disease-specific mortality across cancers. In previous work [5] we formulated the predicted mortality reduction under a stage-shift model and showed it was a function of factors that vary across cancer types, namely, the fraction diagnosed in late stage in the absence of screening and the survival rates for early-versus late-stage diagnoses. Based on this formulation, we predicted that a 10% reduction in late-stage ovarian cancer cases would reduce disease-specific mortality by 6%, but the same reduction in late-stage pancreatic cancer cases would reduce disease-specific mortality by only 1%, due to the markedly worse survival among late-stage pancreatic cancers.

A second caveat is that the standard stage-shift model ignores the distribution of disease subtypes even though they may be quite different for cases diagnosed in late versus early stage. For example, ovarian cancer can be broadly classified into two histologic subtypes (Types I and II). Type II cancers are high-grade serous cancers that make up the majority of late-stage diagnoses and have worse prognosis than the less aggressive Type I cases which are more prevalent among early-stage diagnoses (Table 1, Box 1). Similarly, prostate cancer can be categorized into low-grade (WHO grades I-II) and high-grade (WHO grades III-IV) cancers, with high-grade cancers more likely to be diagnosed in late stage (Table 2, Box 1). Histologic subtypes with differing stage distributions and survival have been identified for many cancers.

Because the standard stage-shift model ignores disease subtypes, early-stage survival (with its mixture of subtypes) is substituted for late-stage survival (with its own mixture of subtypes). This may or may not be plausible biologically; assuming it is the case may affect the accuracy of mortality predictions. Our formulation of the predicted mortality reduction under the stage-shift model [5] ignored disease subtypes.

To accommodate subtypes, we predict mortality reductions separately by subtype, given subtype-specific reductions in late-stage incidence, then sum the results across subtypes. This stratified approach ensures that late-stage cases are shifted to early stage in proportion to the subtype distribution in late stage and therefore does not adopt the mixture of early-stage subtypes without screening. While this approach can be applied in the setting of multiple subtypes, we present examples for the case of two subtypes, comparing predictions from the stratified approach preserving subtype with those from the standard approach in ovarian cancer (Type I and Type II) and prostate cancer (low and high grade). The results suggest general conditions under which stage-shift models that preserve subtype may be preferred.

The predicted ovarian cancer mortality reduction from a screening trial with a hypothetical 30% reduction in late-stage disease is shown in Table 1. The inputs (Box 1) were derived from a study of ovarian cancer incidence and survival based on data from the Surveillance, Epidemiology, and End Results (SEER) database [6]. The mortality prediction (Box 2) uses the stage-shift formulation derived from these inputs, aggregated across subtypes and separately by subtype. The predicted mortality reduction ignoring subtypes is optimistic (3 percentage points or 15% higher) compared to the predicted reduction that preserves subtypes.

The predicted prostate cancer mortality reduction from a screening trial with a hypothetical 30% reduction in late-stage disease is shown in Table 2. Box 1 inputs were derived from the SEER database (RRID:SCR_006902) using cases diagnosed in 1980-1984, prior to dissemination of prostate-specific antigen screening and new, curative treatments. The predicted prostate cancer mortality reduction ignoring grade is again optimistic (3 percentage points or 31% higher) compared to the predicted reduction that preserves subtypes.

We infer from these results that when aggressive subtypes are more prevalent among late-stage diagnoses, a stage-shift model that ignores subtype will be optimistic and could inflate expectations of mortality benefit. This is because the model that ignores subtype assumes that when late-stage cases (which tend to belong to the more aggressive subtype) are shifted to early stage, they receive the average early-stage survival (which is dominated by the less aggressive subtype). In practice, the difference between the predictions of the two models will be moderated by the relative prevalence of the different subtypes and could be very modest if aggressive subtypes are relatively rare.

The ovarian cancer and prostate cancer examples differ in that the ovarian cancer subtypes represent two histologically distinct subtypes, whereas there is some uncertainty as to whether some low-grade prostate cancers might progress to higher grades. If this is the case, then advancing the time of diagnosis via screening may alter grade as well as stage, and the expected result of a stage shift due to screening may be closer to the results obtained ignoring subtype. For prostate cancer, therefore, it might be more accurate to consider the two results, ignoring and accommodating grade, as bracketing the likely predicted mortality reduction from a 30% reduction in late-stage disease.

In practice, the distribution of late-stage cases shifted to early stage may differ from the subtype distribution in late stage in the absence of screening. When information is available on the subtype distribution of late-stage cases with and without screening, we recommend that the implied distribution of subtypes among cases shifted out of late stage be preserved when predicting the reduction in mortality. This approach should provide a more biologically defensible and more accurate assessment of the likely outcome of a novel screening test than the standard stage-shift model.

## Figures and Tables

**Table 1:** Projection of Ovarian Cancer Mortality Reduction in a Hypothetical Screening Trial Ignoring and Preserving Subtypes According to their Distribution Among Late-Stage Diagnoses

Box 1: Inputs to Mortality Reduction Projection
	Type I	Type II	Total
(A) Years of follow-up			10
(B) Annual Incidence Hazard			0.001
(C) Distribution of Cases			
Early Stage	26%	13%	39%
Late Stage	10%	52%	61%
Total	35%	65%	100%
(D) Annual Mortality Hazard			
Early Stage	0.016	0.041	0.024
Late Stage	0.125	0.174	0.165
Total	0.037	0.133	0.090


Box 2: Mortality Reduction Projection
Type I	Type II	Total
(E) Stage Shift Multiplier	0.610	0.597	0.690
(F) Stage Shift	5%	25%	30%
Mortality Reduction			
(G) Projected Ignoring Type			21%
(H) Projected Preserving Type	3%	15%	18%
(I) Absolute Difference			+3%
(J) Relative Difference			+15%

(A) Years of follow-up for hypothetical trial (B) Annual incidence hazard of ovarian cancer estimated from SEER (18 registries, Nov 2020 submission) for women ages 50-54 years in 2000-2009 (C) Distribution of cases from Pavlik et al. [6] (D) Hazard of mortality following diagnosis, derived from 100-month survival rates presented in from Pavlik et al. [6], assuming a constant hazard of mortality. (E) Stage-shift multipliers calculated as in Owens et al. [5], see formulas G and H in that text. The inputs to these values are the length of follow-up (A), overall incidence rate of the disease (B), the rates at which the disease is diagnosed in each stage (C), and stage-specific survival rates (D). The calculation is performed with all subtypes combined, and then separately by subtype using the corresponding inputs. This value is used below to convert a percent reduction in late-stage disease (F) to a percent reduction in disease-specific mortality (G, H). (F) Percent reduction in late-stage cases. Hypothetical value of 30% in screening arm, allocated between subtypes proportional to late-stage case mix (C). (G) = (E) x (F) in total (H) = (E) x (F) for each subtype separately and summed across subtypes (I) and (J) Absolute and relative differences of projection in total compared to projection by subtype

**Table 2:** Projection of Prostate Cancer Mortality Reduction in a Hypothetical Screening Trial Ignoring and Preserving Subtypes According to their Distribution Among Late-Stage Diagnoses

Box 1: Inputs to Mortality Reduction Projection			
	Low Grade	High Grade	Total
(A) Years of follow-up			10
(B) Incidence Hazard			0.005
(C) Distribution of Cases			
Early Stage	66%	13%	80%
Late Stage	8%	13%	20%
Total	74%	26%	100%
(D) Mortality Hazard			
Early Stage	0.017	0.087	0.026
Late Stage	0.161	0.258	0.209
Total	0.026	0.138	0.045


Box 2: Mortality Reduction Projection			
	Low Grade	High Grade	Total
(E) Stage Shift Multiplier	0.399	0.292	0.437
(F) Stage Shift	11%	19%	30%
Mortality Reduction			
(G) Projected Ignoring Grade			13%
(H) Projected Preserving Grade	5%	5%	10%
(I) Absolute Difference			+3%
(J) Relative Difference			+31%

(A) Years of follow-up for hypothetical trial (B) Incidence hazard of prostate cancer estimated from SEER (9 registries, Nov 2004 submission) for men ages 50-59 years in 1980-1984 (C) Distribution of cases from SEER for men diagnosed ages 50-59 years in 1980-1984 (D) Hazard of mortality following diagnosis, derived from SEER 10-year survival rates, assuming a constant hazard of mortality. (E) Stage-shift multipliers calculated as in Owens et al. [5], see formulas G and H in that text. The inputs to these values are the length of follow-up (A), overall incidence rate of the disease (B), the rates at which the disease is diagnosed in each stage (C), and stage-specific survival rates (D). The calculation is performed with all grades combined, and then separately by grade using the corresponding inputs. This value is used below to convert a percent reduction in late-stage disease (F) to a percent reduction in disease-specific mortality (G, H). (F) Percent reduction in late-stage cases. Hypothetical value of 30% in screening arm, allocated between grades proportional to late-stage case mix (C). (G) = (E) x (F) in total (H) = (E) x (F) for each grade separately and summed across grades (I) and (J) Absolute and relative differences of projection in total compared to projection by grades

## Data Availability

The incidence and survival data analyzed in this study are available from the SEER website at https://seer.cancer.gov/data-software.
